# Bench surgery with autotransplantation for bilateral Wilms tumor—A feasible technique for renal sinus invasion

**DOI:** 10.3389/fsurg.2022.1047975

**Published:** 2022-12-13

**Authors:** Pengfei Gao, Jun Li, Huadong Chen, Wenrui Wu, Longshan Liu, Hong Jiang, Lingling Xu, Chenglin Wu, Qian Fu, Juncheng Liu, Changxi Wang

**Affiliations:** ^1^ Organ Transplant Center, The First Affiliated Hospital, Sun Yat-sen University, Guangzhou, China; ^2^ Department of Pediatric Surgery, The First Affiliated Hospital, Sun Yat-sen University, Guangzhou, China; ^3^ Pediatric Intensive Care Unit, The First Affiliated Hospital of Sun Yat-sen University, Guangzhou, China; ^4^ Guangdong Provincial Key Laboratory of Organ Donation and Transplant Immunology, The First Affiliated Hospital, Sun Yat-sen University, Guangzhou, China; ^5^ Guangdong Provincial International Cooperation Base of Science and Technology (Organ Transplantation), The First Affiliated Hospital, Sun Yat-sen University, Guangzhou, China

**Keywords:** bilateral Wilms tumor, bench surgery, autotransplantation (AT), nephron-sparing surgery (NSS), renal sinus invasion

## Abstract

**Purpose:**

Bilateral Wilms tumor (BWT) with renal sinus invasion requires extremely difficult surgical care. This study presents an alternative strategy for tumor removal while at the same time preserving the renal parenchyma.

**Materials and methods:**

In total, 9 cases of synchronous BWT were admitted to our hospital between May 2016 to Aug 2020. We retrospectively reviewed the clinical data, surgical technique, and functional and oncological outcomes of these cases.

**Results:**

The 9 cases included 3 males and 6 females, with a median age of 12 months at surgery (range 7–40). A total of 14 kidney units had renal sinus invasion (77.8%), whereas multifocal neoplasms were observed in 7 units (38.9%). The local stage distribution revealed 1 kidney with stage I, 10 kidneys with stage II, and 7 kidneys with stage III. Nephron-sparing surgery was performed on 15 kidney units (83.3%), among which 13 (72.2%) underwent bench surgery with autotransplantation (BS-AT), whereas 2 (11.1%) were subjected to tumor enucleation *in vivo*. Urinary leakage was the most prevalent postoperative complication. We observed negative margins. During the mean follow-up of 28.4 months, 2 patients (22.2%) succumbed from sepsis and renal failure, respectively, whereas the other 7 (77.8%) survived without recurrence. Survivors experienced an estimated glomerular filtration rate of 81 ± 15.4 ml/(min × 1.73 m^2^). The endpoint renal volume of 9 renal units receiving BS-AT significantly increased (*P* = 0.02).

**Conclusions:**

In summary, the surgical management of bilateral Wilms tumor requires meticulous operative approach and technique. Besides, BS-AT provides a viable alternative to nephron-sparing surgery for BWT patients with renal sinus invasion.

## Introduction

Wilms tumor is the most prevalent pediatric kidney tumor originating from undifferentiated embryonic lesions. Bilateral Wilms tumor (BWT) accounts for 4%–13% of Wilms tumor cases, among which 65% are synchronous BWT (1, 2). Patients with synchronous BWT are at greater risk of developing renal failure. The potential risk factors include recurrent tumor, intrinsic renal disease, inadequate renal parenchyma, predisposition syndromes, and chemotherapy- and/or radiotherapy-induced nephrotoxicity (3–5).

Synchronous BWT presents a significant challenge for clinicians. Based on a surgical standpoint, there is a delicate balance between tumor removal and preservation of renal function. The most widely used surgical modality is nephron-sparing surgery (NSS), allowing patients to forego renal replacement therapy (6). Ideal candidates are patients with a unifocal mass in the upper or lower pole of the kidney, sparing at least a third of the kidney, no signs of metastases or renal sinus invasion, and favorable histology (7). Nonetheless, surgeons are impressed by the prevalence of renal sinus invasion and multifocal lesions in BWT patients (8, 9). Generally, NSS is performed *in vivo* using an open transperitoneal approach, which intraoperatively increases the promising rate of tumor rupture and incomplete resection (10).

Tumor resection *ex vivo* using bench surgery with autotransplantation (BS-AT) is the ultimate approach to NSS (11). In 1975, John first described BS-AT, as a hypothesis (12). Only a limited minority of BWT patients underwent BS-AT in the past few decades (8, 13). To our knowledge, comprehensive and specific strategy for BS-AT and management of postoperative complications remain unreported. This work described an experience of surgical management of synchronous BWT, particularly the use of BS-AT.

## Materials and methods

### General data

In this retrospectively study, we reviewed the clinical data of 9 patients with synchronous BWT admitted to the First Affiliated Hospital of Sun Yat-sen University between May 1, 2016, to Aug 30, 2020. This study was approved by the Institutional Review Board of the First Affiliated Hospital of Sun Yat-sen University (IRB-2021-129). Informed patient consent was waived due to its retrospective nature.

Among the 9 patients, 3 were male and 6 were female. Case 1 had multiple anomalies (hemihypertrophy, hypospadias and cryptorchidism), whereas case 2 had Denys-Drash syndrome (DDS). Pulmonary nodules were observed in case 4 but disappeared after preoperative chemotherapy. Case 7 had an indirect hernia. Case 8 had a thrombus in the right renal vein and inferior vena cava. Case 9 had WAGR (Wilms tumor, Aniridia, Genitourinary abnormalities, intellectual disability) syndrome with other ocular anomalies (cataract and glaucoma). Table 1 presents the clinical data of the 9 patients. Only 2 patients did not receive any treatment before reaching our hospital, prehospital managements for other 7 patients varied. Except for case 2, biopsies were performed on 8 patients before preoperative chemotherapy. All patients were treated with 2-drug preoperative chemotherapy (vincristine and actinomycin D), 2 with additional doxorubicin (cases 1 and 8), and 3 with additional carboplatin and etoposide (cases 1, 2, and 5). The tumors had varying degrees of shrinkage. Postoperative chemotherapy was administered according to the SIOP 2016 chemotherapy protocol guidelines based on the local stage and pathological results. None of the patients had received radiotherapy.

**Table 1:** Clinical characteristics of 9 patients.

Case	Gender	Age at surgery (months)	Associated anomalies or thrombus	Stage (L/R)	Renal hilum involved (L/R)	Renal pelvis invaded (L/R)	blood loss (L/R) (ml)	Type of surgery (L/R)	Histology	Margin	Complication
**Group 1: Staged surgery**											
1	M	12	Hemihypertrophy, hypospadias, cryptorchidism	II/II	−/+	+/+	20/30	BS-AT/BS-AT	Necrosis, epithelial	–	Urinary leakage, hypertension
2	F	9	DDS	II/II	−/−	−/−	20/20	BS-AT/tumor enucleation *in vivo*	Mixed	–	Urinary leakage, hypertension
3	F	7	–	II/II	+/+	+/+	40/35	RN/BS-AT	Necrosis, mixed	–	Urinary leakage, chylous ascites hydronephrosis
4	M	40	–	III/III	+/+	+/+	30/10	BS-AT/RN	Necrosis, mixed	–	–
5	F	15	–	III/III	+/+	−/−	50/30	BS-AT/BS-AT	Mixed, NRs	–	–
6	F	8	–	III/II	+/+	+/+	150/20	BS-AT/BS-AT	Necrosis, mixed	–	hydronephrosis
**Group 2: Simultaneous surgery**											
7	M	10	Indirect hernia	III/III	+/+	−/−	200/100	BS-AT/BS-AT	Epithelial, NRs	–	DIC, sepsis
8	F	34	Thrombus in the renal vein and vena cava	II/II	+/+	+/+	80/20	BS-AT/RN	Necrosis, stromal	–	AKI, urinary leakage, hypertension
9	F	13	WAGR, cataract, glaucoma	II/I	+/−	+/−	40/60	BS-AT/tumor enucleation *in vivo*	Necrosis, stromal,	–	–

RN, radical nephrectomy; ESRD, end stage renal disease; DIC: disseminated intravascular coagulation; BS-AT: bench surgery with autotransplantation; AKI: acute kidney injury; WAGR: Wilms tumor, Aniridia, Genitourinary abnormalities, intellectual disability.

Pre-operative evaluations included urological ultrasonography, computerized tomography (CT), and renal dynamic radionuclides. The Schwartz formula was used to evaluate the estimated glomerular filtration rate (eGFR). The absolute renal volume was calculated using the formula for a prolate ellipsoid (max length × max width × max depth × 0.532) (14). Renal function was monitored using serum creatinine, CT, and renal dynamic radionuclides.

### BS-AT technique

**Figure 1:**
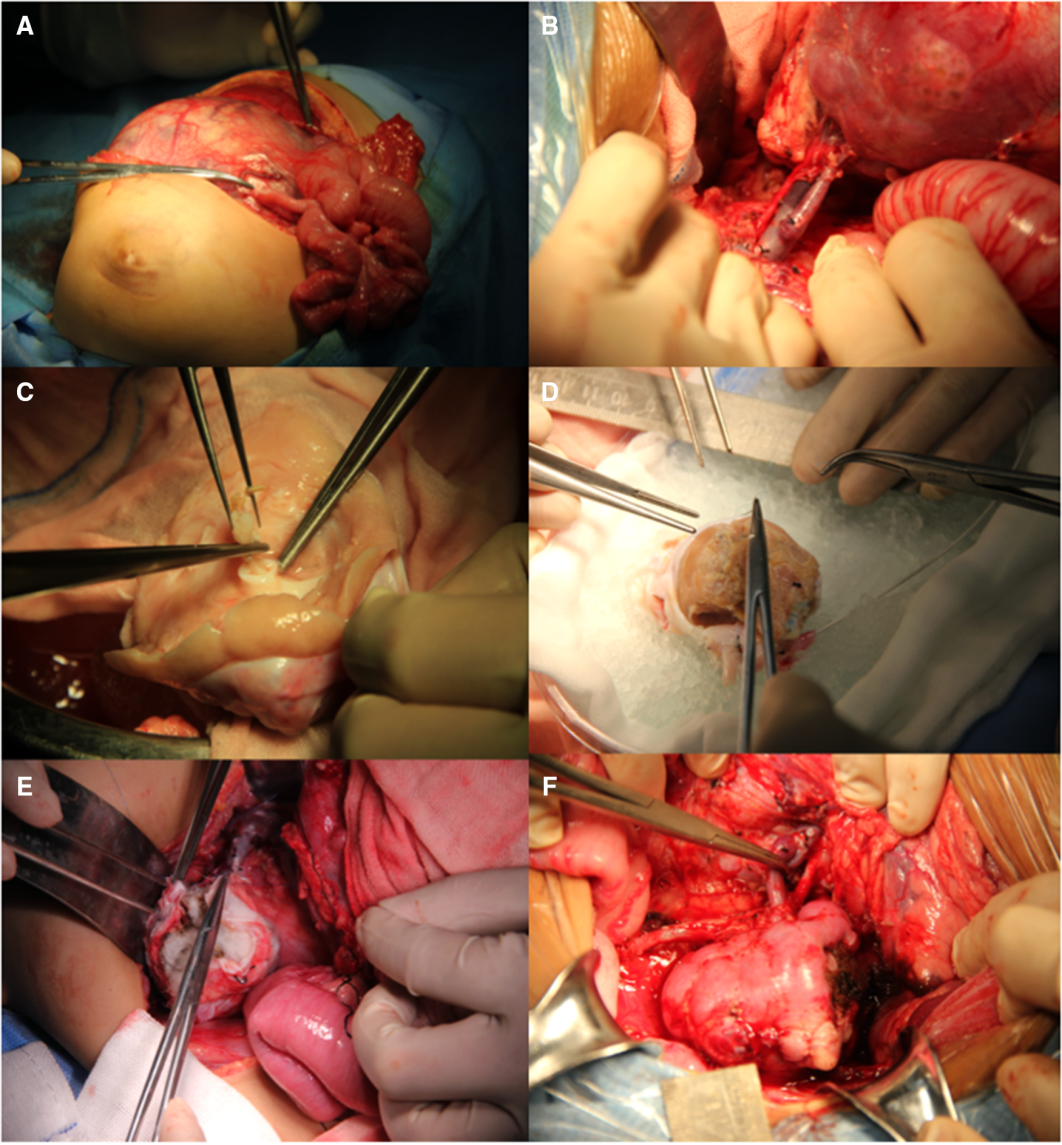
Surgical procedure of BS-AT. (**A**) Transverse upper abdominal incision. (**B**) Renal pedicle was well exposed. (**C**) Tumor removal in the renal pelvis. (**D**) Residual renal parenchyma and renal pelvis system were reconstructed with cooling perfusion. (**E**) Hemostatic sponge was used to cover the residual kidney. (**F**) Orthotopic kidney transplantation with vascular and ureteral anastomoses.

This procedure involves radical nephrectomy (RN), *ex vivo* tumor removal with bench surgery, and kidney autotransplantation. A transverse upper abdominal incision was made to completely expose the tumor. Notably, damage to blood vessels and tumor rupture should be avoided in the process of RN. Tumors were marked with electric coagulation before extracting the involved kidney. The renal vessels were set aside for approximately 1.0 cm–1.5 cm, and the ureter for approximately 4.0 cm–5.0 cm. The kidney was immediately transferred to a workbench and perfused with hypertonic citrate adenine (HCA) solution. Neoplasms were exposed after sharply opening the capsule. Tumor excision was performed using a scalpel or ultrasonic scalpel, and the margins of the residual kidney were sent for frozen section examination. The renal pelvis was opened in cases of tumor invasion. Hemorrhage and leakage were detected and treated in the process of bench surgery. Non-absorbable sutures were used to repair the remaining kidney parenchyma, whereas absorbable sutures were used to reconstruct the renal pelvis. Subsequently, a double-J tube was inserted into the anastomosed ureter. Thereafter, the remaining kidney was orthotopically transplanted and covered with a hemostatic sponge. Urine output was monitored following vascular and ureteral anastomoses. During the entire procedure, the kidneys were placed on ice to maintain hypothermia (Figure 1). Enoxaparin sodium (1000 U, qd, 3–5 days) and subsequently followed by clopidogrel (12.5 mg, qd, 1 month) were applied postoperatively.

### Statistical analysis

Descriptive statistics appropriately included mean ± SD, median with continuous variables, and percentages as appropriate. The student's *t*-test was used for comparisons between continuous variables. Statistical analysis was performed using the SPSS version 26.0 software for Macs.

## Results

The median age at the time of surgery was 12 months (range, 7–40 months), whereas the mean interval between neoadjuvant chemotherapy and surgery was 3.4 ± 1.6 months. The local stage distribution revealed: 1 kidney in stage I, 10 kidneys in stage II, and 7 kidneys in stage III.

A total of 6 patients (cases 1–6) underwent staged operations, whereas 3 patients (cases 7–9) underwent a single-stage operation. Among the 18 renal units, 14 kidneys (77.8%) had renal sinus invasion, including 14 with hilar involvement and 11 with renal pelvic invasion. Multifocal neoplasms were observed in 7 kidney units (38.9%).

**Figure 2:**
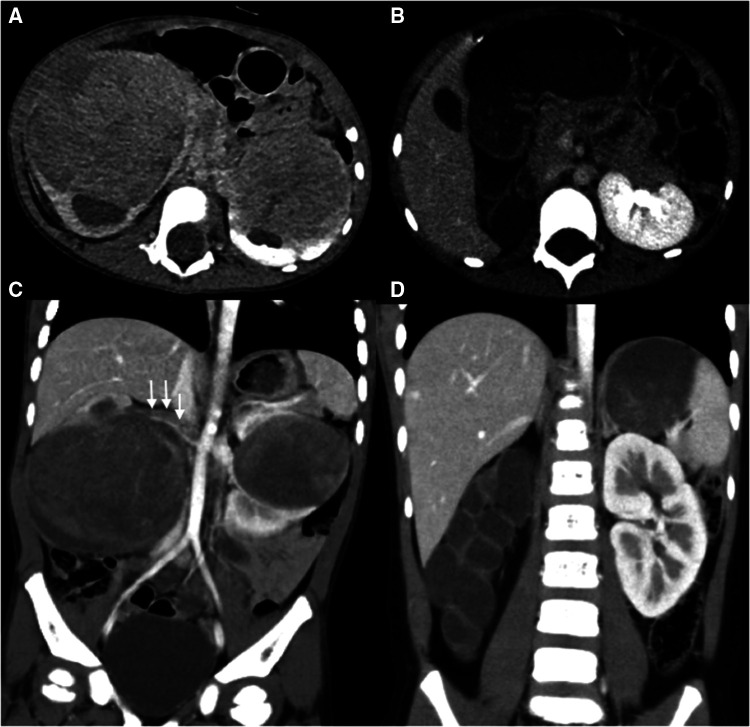
Abdominal CT scans of case 8. (**A,C**) Preoperative CT images reveal bilateral kidney lesions invading the renal sinus with bilateral hydronephrosis, a filling defect is noted within the right renal vein and inferior vena cava (white arrow). (**B,D**) Postoperative CT images reveal the absence of right kidney and Normal left morphology.

**Figure 3:**
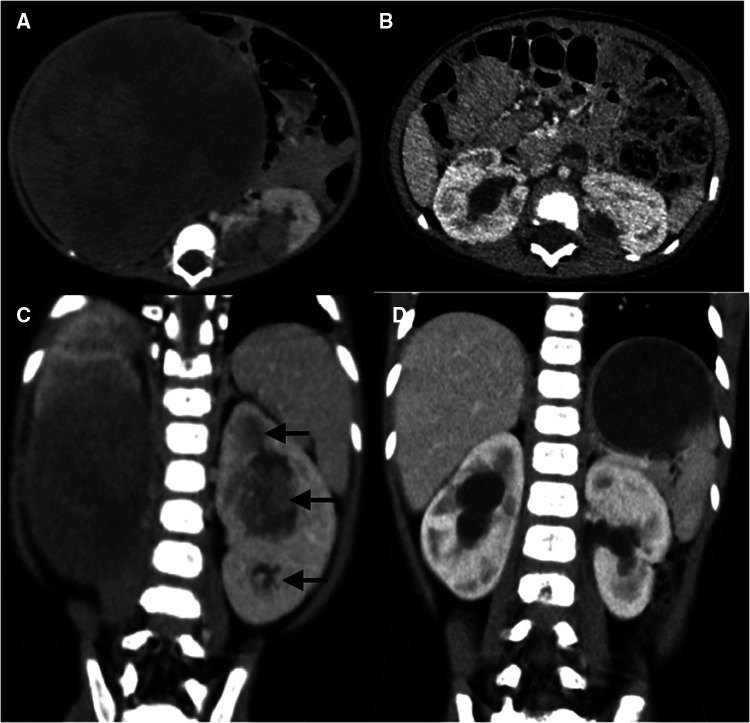
Abdominal CT scans of case 6. (**A,C**) Preoperative CT images reveal a huge renal mass in the right kidney and multifocal neoplasms in the left kidney (black arrow), with bilateral renal sinus invasion. (**B,D**) Postoperative CT images reveal multiple perfusion defects in both kidneys and bilateral mild hydronephrosis, without evidence of recurrence or metastasis.

**Figure 4:**
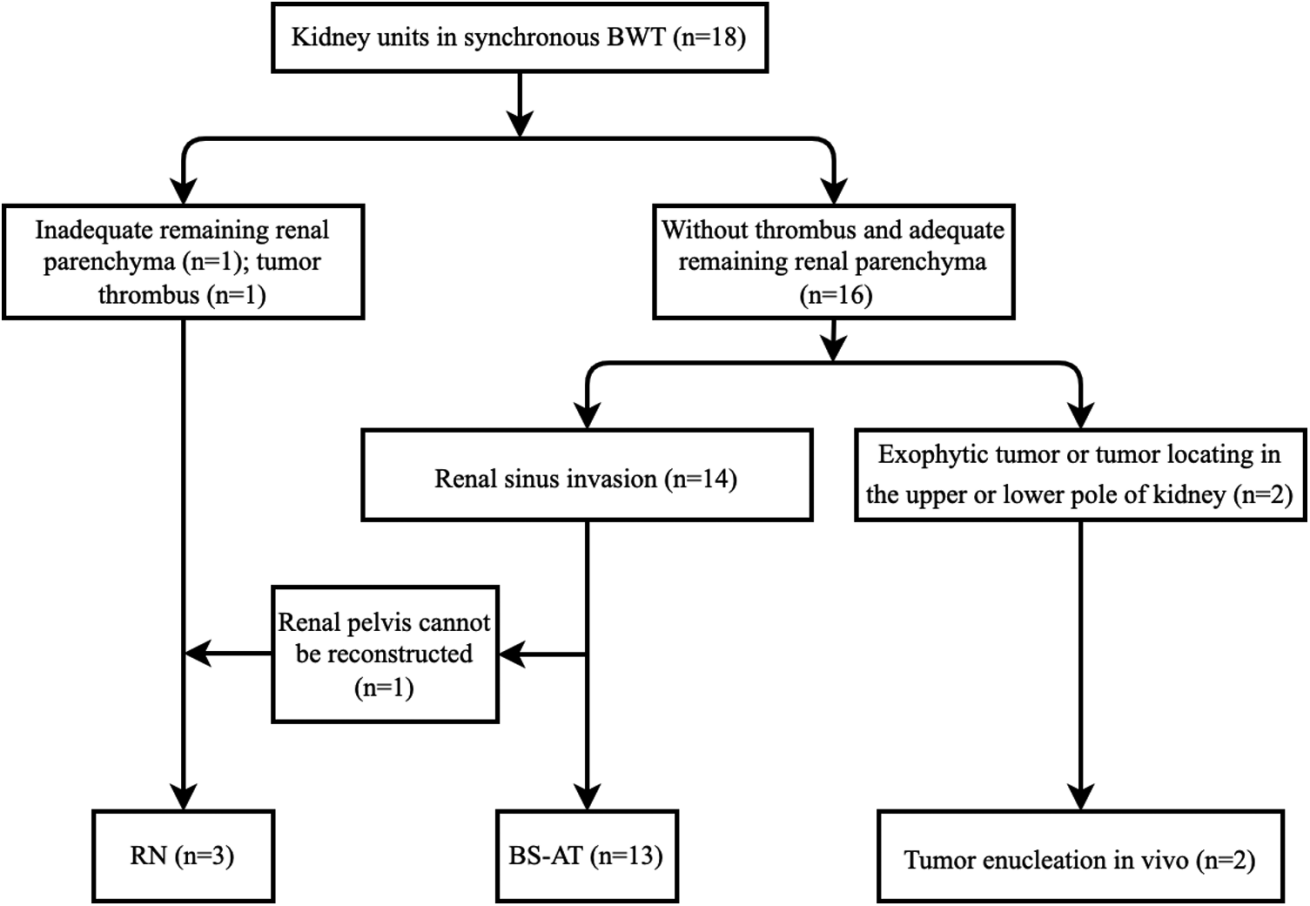
Flow chart of surgical strategy.

A total of 3 patients (cases 3, 4, and 8) received one side NSS and contralateral RN, whereas the other 6 patients received bilateral NSS. RN was performed in 3 kidney units due to the absence of adequate renal tissue, tumor thrombus, and unsuccessful reconstruction of the renal pelvis. In total, 15 kidney units received NSS, 13 underwent BS-AT, and 2 underwent tumor enucleation *in vivo*, accounting for 83.3%, 72.2%, and 11.1%, respectively (Figures 2, 3). Figure 4 shows the surgical approach. The median blood loss on each side was 32.5 ml (range 10–200 ml), and 3 patients required blood transfusion (cases 6–8).

The pathological results revealed favorable histological types in all children, and we observed negative margins. Necrosis was observed in 6 patients, whereas nephrogenic rests were noted in 2 patients (cases 5 and 7). The histology of the residual kidney in case 2 showed diffuse nephritis with mesangial sclerosis.

A total of 4 patients experienced urinary leakage, adequate drainage with ultrasound-guided percutaneous nephrostomy resolved this problem; transplanted renal artery stenosis was not observed in the postoperative CT scans; 3 children had postoperative hypertension (within 3 months), for effective oral antihypertensive drugs, and oral antihypertensive drugs were discontinued when blood pressure normalized; 2 patients had chylous ascites, whereas 1 developed perinephritis. The disorder was appropriately resolved using drainage and antibiotics. Case 8 developed acute kidney injury (AKI) after surgery and necessitated hemodialysis for 2 weeks until the renal function improves.

**Figure 5:**
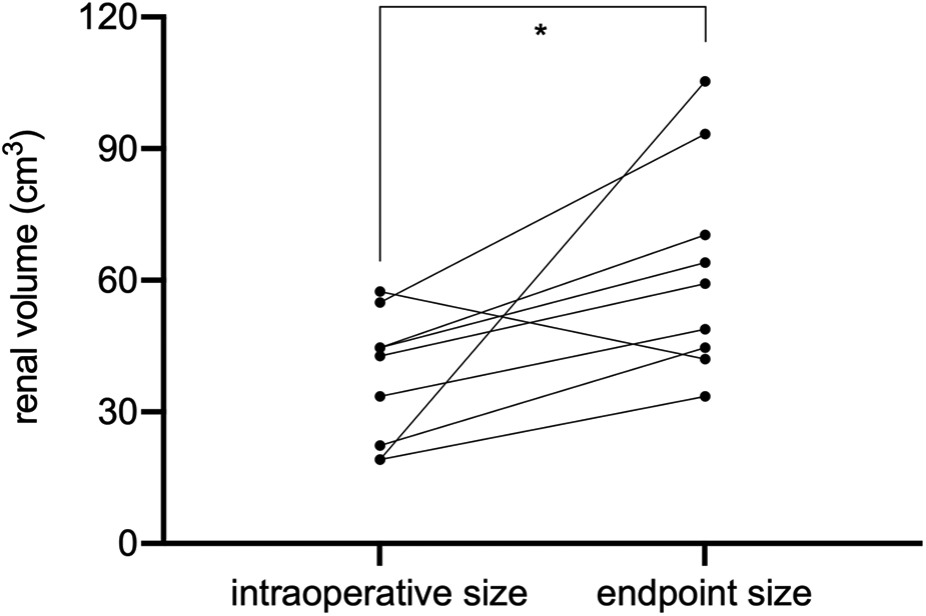
Renal volume at surgery and follow-up endpoint (*P = 0.02).

The endpoint of follow-up was May 09, 2022. All patients were followed up for an average of 28.4 ± 16.1 months; 2 children died due to end-stage renal disease (ESRD) (case 1) and postoperative sepsis (case 7), respectively (Table 2); 7 children survived and finished postoperative chemotherapy with no evidence of tumor recurrence. Case 1 had an eGFR of less than 50 ml/(min × 1.73 m^2^) preoperatively and rapidly developed renal insufficiency after surgery. Case 2 had an eGFR higher than 80 ml/(min × 1.73 m^2^) 2 months after surgery, but developed ESRD 11 months postoperatively and was eventually subjected to allograft renal transplantation 28 months after BS-AT. No statistically significant differences were noted in eGFR after 2 weeks, 1 month, and 3 months postoperatively compared to the preoperative levels (*P* > 0.05). However, the follow-up endpoint eGFR decreased significantly from preoperative levels (*P* = 0.048). Nevertheless, the mean eGFR of survivors was 81 ± 15.4 ml/(min × 1.73 m^2^). In our cohort, 11 renal units survived, 9 of which were subjected to BS-AT. In these 9 units, the intraoperative renal volume was 37.63 ± 14.79 cm^3^, whereas the follow-up endpoint renal volume was 62.38 ± 24.05 cm^3^, with a statistical difference (*P* = 0.02) (Figure 5; **Supplementary Table 1**).

**Table 2:** Functional and oncological outcomes of 9 patients.

Case	eGFR (ml/min/m^2^)					Follow-up (m)	Outcome
	Preop*	Postop^#^ (2 weeks)	Postop^#^ (1 month)	Postop^#^ (3 months)	Postop^#^ (endpoint)		
1	43	19	13	7	6	4	Death, ESRD
2	114	98	129	66	4	36	Alive with allograft KT
3	71	82	70	82	83	40	Alive
4	91	101	91	88	89	38	Alive
5	114	79	70	78	77	47	Alive
6	185	161	127	210	122	23	Alive
7	129	41	–	–	41	1	Death, sepsis
8	105	10	52	119	66	35	Alive
9	161	156	175	203	127	32	Alive

ESRD, end stage renal disease; eGFR, estimated glomerular filtration rate; KT, kidney transplantation.

*The preoperative eGFR was presented before the first surgery in case 1–6.

^#^ The postoperative eGFR was presented after the second surgery in case 1–6.

## Discussion

Proper surgical techniques are the mainstay of multimodal therapies that improve oncologic outcomes for BWT. The available options for synchronous BWT include unilateral RN with contralateral NSS, bilateral NSS, or bilateral RN, and the most common surgical procedure is unilateral RN with contralateral NSS (1). Drysdale reviewed BWT from 4 centers and discovered that unilateral RN with contralateral NSS was the preferred option in low-income centers, whereas bilateral NSS was more prevalent in high-income centers (15). Presumably, patients in low-income centers may have a higher stage distribution. Herein, 94.4% of renal units were distributed in stage II or stage III, 6 cases (66.7%) received bilateral NSS, and 61.1% of renal units were preserved without recurrence. Despite the small number of cases, our results were encouraging. Figure 4 shows our surgical strategy. Tumor enucleation *in vivo* was performed if neoplasms located at the periphery or polar region; RN was performed in cases of thrombus or absence of adequate renal parenchyma. Meanwhile, RN remains inevitable if the renal pelvis cannot be reconstructed following tumor eradication. We admitted BS-AT increases the surgical difficulty, but it contributes rescuing more renal units as the ultimate approach to NSS (11). Notably, Our study indicated that BS-AT is a practical option in cases of renal sinus invasion.

In order to contribute the renal preservation, preoperative chemotherapy is used to promote tumor regression. Increased utilization of preoperative chemotherapy could further expand the number of patients eligible for partial nephrectomy (16). In the SIOP 2016 protocol, NSS is the preferred option in BWT patients. If NSS seems impossible, the tumors could be biopsied, preferably with the Tru-cut needle. In non-responsive to preoperative chemotherapy BWT patients, WT1 mutations or unfavorable histology need to be ruled out. A biopsy should be performed to confirm histology to decide further treatment (17–19). Meticulous assessment of renal function is necessary to substantially renal parenchyma in synchronous BWT. Preoperative imaging helps in delineating the extent of kidney involvement. Besides the spatial location of the tumor, the vascular supply and renal sinus must have to be thoroughly assessed. After surgery, most bilateral cases often develop varying degrees of renal insufficiency (20). A similar observation was made in our series. Although postoperative eGFR within 3 months was not statistically different from preoperative, the follow-up endpoint of eGFR was statistically decreased. Perhaps, the remaining renal parenchyma was at increased risk of glomerulosclerosis and progressive renal failure (21). The decreased renal volume also contributes to the eventual ESRD and decreased GFR (5, 14). Case 1 suffered preoperative renal impairment [eGFR less than 50 ml/(min × 1.73 m^2^)], which was considered to be associated with intrinsic renal disease and nephrotoxic chemotherapy. The renal function of case 1 rapidly deteriorated after the staged surgery, this case suggested that neoadjuvant chemotherapy should not trigger nephrotoxicity before BS-AT. Since the tumor size is unlikely to shrink further and postoperative chemotherapy depends on surgical pathology, neoadjuvant chemotherapy should not last more than 12 weeks (17, 22). In our study, BS-AT was performed within 1 month after the completion of preoperative chemotherapy. Because of DDS, case 2 showed dramatic deterioration of renal function 11 months postoperatively. During this time-lapse, the patient completed postoperative chemotherapy, and tumor recurrence was not observed. Allograft kidney transplantation is advised to be performed 2 years after the completion of chemotherapy (23). Given the possibility of tumor recurrence and chemotherapy-related nephrotoxicity, allograft kidney transplantation is not recommended in conjunction with an initial bilateral nephrectomy. At the same time, allograft kidney transplantation is not necessary for patients with good renal function after BS-AT.

Herein, tumor size did not play a significant role in surgical planning. Despite the massive tumor in the right kidney of case 6 (Figure 3), the remaining renal parenchyma measured 44.69 cm^3^, and we successfully performed NSS using BS-AT. Kubiak et al. discovered that favorable postoperative renal function can be anticipated if at least 30% of the renal parenchyma is preserved (24). The minimum renal volume after tumor removal in our study was 16.76 cm^3^, and the volume of surviving renal units significantly increased at the follow-up endpoint. This change could be explained by hypertrophy of the remaining nephrons because of an increase in glomerular capillary pressure and flow, which serves to minimize renal functional loss (21). Also, we noted that among the surviving renal units, the volume remained unrestored after tumor enucleation *in vivo*. We proposed the following explanations: a severely reduced number of nephrons will probably not compensate *via* hypertrophy (14), and tumor resection *in vivo* causes more severe thermal ischemia-reperfusion injury.

Brilliant surgical skills and cooperation among transplant surgeons and pediatric surgeons are vital for the successful implementation of BS–AT. Notably, it is difficult to discriminate tumor tissue from normal kidney tissue after perfusion (25). Visible tumors should be marked before RN. Complete tumor excision and a margin of the normal renal parenchyma are still recommended (26). Repeated frozen sections in bench surgery can be performed to achieve negative margins. At our Organ Transplant center, ice-cold HCA solution is routinely used to perfuse donor kidneys to alleviate ischemia-reperfusion injury. A similar study revealed that vascular clamping during tumor excision *in vivo* took no more than 30 min (20). Nonetheless, bench surgery allows surgeons sufficient time to clarify the spatial relationship between the tumor and renal sinus, resect the tumor, and perform retroperitoneal lymph node sampling. To ensure patient safety, the renal vein was ligated when the kidney was taken out. Surgical members paid enhanced attention to secondary bleeding. A vascular clamp was used for blocking the proximal renal vein or partial occlusion of vena cava.

Individualized surgical procedures should be investigated, including: (1) simultaneous or staged operation; (2) which kidney is the priority for treatment. Warmann et al. recommended single-stage surgery, and tumor resection should be started on the more affected side; the contralateral kidney should be first treated in patients when NSS is unfeasible (27). In our series, the staged surgery appeared to alleviate surgical trauma and intraoperative blood loss, AKI was not present. Considering the subsequent contralateral RN, we first performed NSS on the less involved side.

Several early complications are linked to nephrectomy, such as intestinal obstruction, extensive hemorrhage, wound infection, and vascular damage (28). In this study, the most prevalent postoperative complication was urinary fistula, specifically in patients with renal pelvic involvement. Consequently, we recommend efficient drainage insertion and meticulous reconstruction of the renal collecting systems. Besides the Double-J tube in the ureter, drainage tubes were placed in the perirenal and pelvic cavities to guarantee adequate drainage. Refractory urinary fistulas are managed *via* a percutaneous puncture or retrograde intubation. Absorbable barbed sutures are widely used in adult NSS given the renal growth capacity. In our study, we used non-absorbable sutures as we were concerned about secondary bleeding after BS-AT. We observed that renal volume of 9 renal units receiving BS-AT significantly increased. With the accumulation of surgical experience, absorbable sutures can be attempted in future works. Absorbable sutures are recommended as a precaution against urolithiasis, and additives of papaverine as well as hemostatic cotton to prevent vascular spasms and deformation. We observed no postoperative vascular embolization or transplanted renal artery stenosis. Case 7 highlighted the importance of anti-infective treatment after the implementation of BS-AT. In order to avoid aggravation of postoperative infection, anti-infection therapy should be intensified after BS-AT. Furthermore, for patients with bilateral reconstruction of renal collection system, staged procedures seemed to be safer.

Considering that this was a retrospective study involving a small sample size, there is a need for additional comparable data from multiple centers and a longer follow-up period. As a feasible technique that preserves the renal parenchyma, BS-AT is expected to be applied in patients with one solitary kidney, metachronous BWT, or other tumors affecting the renal sinus. A highly experienced team must centralize the surgery for effective treatment of these patients.

## Conclusion

In conclusion, we found that it was technically practical to apply BS-AT in the management of synchronous BWT albeit in our relatively small series. Moreover, patients with renal sinus invasion can benefit from BS-AT, which contributes to achieving a leisurely NSS without compromising tumor control. Therefore, meticulous surgical approaches and excellent skills are paramount to achieving acceptable oncological and functional outcomes.

## Data Availability

The original contributions presented in the study are included in the article/**Supplementary Material**, further inquiries can be directed to the corresponding authors.
