# Supplementary material for: Bench surgery with autotransplantation for bilateral Wilms tumor—A feasible technique for renal sinus invasion
Source: Front Surg. 2022 Dec 13;9:1047975. doi: 10.3389/fsurg.2022.1047975 (PMC9793901; doi:10.3389/fsurg.2022.1047975)
Supplement: Supplementary file 1 [file Table1.docx]

Supplementary table 1: Renal volume at surgery and endpoint

| Case | Intraoperative size (cm)/Vol^*^ (cm^3^) | | Endpoint size (cm)/Vol^*^ (cm^3^) | |
| --- | --- | --- | --- | --- |
|  | L | R | L | R |
| 1 | 6.0×5.0×3.0/47.88 | 6.0×3.0×2.5/23.94 | 5.6×4.8×4.4/62.91 | 4.6×4.0×4.6/45.03 |
| 2 | 8.0×4.5×3.0/57.46 | 8×5.0×5.0/106.40 | 5.7×4.2×3.3/42.03 | 6.7×4.0×3.3/47.05 |
| 3 | - | 4.0×3.0×3.0/19.15 | - | 7.2×5.5×5.0/105.34 |
| 4 | 6.0×4.0×3.5/44.69 | - | 7.0×4.3×4.0/64.05 | - |
| 5 | 4.0×3.0×3.0/19.15 | 4.0×3.0×3.5/22.34 | 5.0×3.6×3.5/33.51 | 6.0×4.0×3.5/44.69 |
| 6 | 6.0×3.5×3.0/33.52 | 6.0×4.0×3.5/44.49 | 6.2×3.7×4.0/48.82 | 6.7×4.7×4.2/70.36 |
| 7 | 3.5×3.0×3.0/16.76 | 4.0×3.0×3.0/19.15 | - | - |
| 8 | 6.0×4.3×4.0/54.90 | - | 7.8×5.0×4.5/93.37 | - |
| 9 | 6.7×4.0×3.0/42.77 | 8.5×4.5×4.0/80.40 | 7.2×4.3×3.6/59.29 | 7.4×4.2×3.5/57.87 |
